# A latent profile analysis of subjective exercise experiences among physically vulnerable college students and psychiatric symptoms correlates during three phases of the COVID-19 pandemic in Wuhan, China

**DOI:** 10.3389/fpsyg.2023.1118489

**Published:** 2023-03-30

**Authors:** Jinglin Li, Ming Xiao, Hongyuan Huang, Huinan Liu

**Affiliations:** ^1^Department of Physical Education, Zhongnan University of Economics and Law, Wuhan, China; ^2^Department of Physical Education, Middle School Attached to Huazhong University of Science and Technology, Wuhan, China; ^3^Department of Special Education and Counselling, The Education University of Hong Kong, Hong Kong, China

**Keywords:** physically vulnerable, college students, subjective exercise experiences, latent profile analysis, depression, anxiety

## Abstract

**Introduction:**

Physical activity among college students since the COVID-19 pandemic was well studied; however, subjective exercise experience and the emotional response toward physical exercise received less attention.

**Methods:**

The present study used latent profile analysis (LPA) to explore the latent class of subjective exercise experience among physically vulnerable college students who scored 59 points or less in tests under the National Student Physical Health Standard. Three non-duplicated samples at different stages of the COVID-19 pandemic were collected in March 2020 (*N* = 127), March 2021 (*N* = 118), and November 2021 (*N* = 206) respectively. Psychometrically validated scales, namely, Subjective Exercise Experiences Scale (SEES), Generalized Anxiety Disorder (GAD-7), and Patient Health Questionnaire (PHQ-9) were used to measure subjective exercise experience, anxiety symptoms, and depressive symptoms.

**Results and discussion:**

LPA revealed a 3-class solution for the subjective exercise experience of physically unfit students, namely, the “negative experience group” (30.82%), the “fatigue group” (41.91%), and the “positive experience group” (27.27%). Multinomial regression showed that probable anxiety [odds ratio (OR) = 0.12] was associated with the overall negative exercise experience while probable depression (OR = 0.19) was associated with psychological fatigue. Women (OR = 0.496) were more likely to experience overall negative exercise experience, and the outbreak of the COVID- 19 (OR = 2.14) pandemic influenced the psychological distress of the subjective exercise experience compared with the other two phases in the post-COVID- 19 era. Our findings provided significant implications for physical education targeting university students that interventions should be tailored differently for three profiles of the subjective exercise experience.

## Introduction

In China, every year, college students at the national level are required to take the National Student Physical Fitness Test, which evaluates body composition [i.e., body mass index (BMI) in kg/m^2^], cardiorespiratory fitness [800-m running (female)/1,000-m running (male)], muscular strengths endurance [numbers of curl-ups (female) and pull-ups (male) completed in 1 min], explosive leg power [distance of a standing long jump (in cm)], the flexibility (sit and reach), velocity (50-m running), vital capacity, and sight (Guo et al., 2019). The problem of college students' physical decline has still not been effectively addressed as reflected by the test results. Nevertheless, for a long time, people have paid more attention to college students' academic performance but have ignored their mental health and physical health. College students who fail the National Student Physical Fitness Test are more likely to be vulnerable to multiple physical (e.g., overweight, obesity, and underweight) and mental health problems (e.g., depressive symptoms, anxiety, and negative affect) that keep them off from participating in physical activities.

Subjective exercise experience is defined as the subjective feeling and emotional response toward exercise after participating in physical exercise, including positive wellbeing, psychological distress, and fatigue dimensions (MeAuley and Courneya, 1994). Subjective exercise experience is an important predictor of actual exercise behavior (Jiang, 2022). The positive effects of physical exercise on psychological wellbeing have been proven in previous research (Kwan and Bryan, 2010). Abundant evidence showed that people with a positive exercise experience can correctly understand the value and significance of exercise, and positive affective response to exercise contributes to adherence to an exercise regimen (Williams et al., 2008). On the contrary, those who have negative exercise experiences will avoid physical exercise practice and hardly form positive and regular exercise behaviors (Wu et al., 2016). Physical exercise is positively correlated with the subjective wellbeing of college students (Shang et al., 2021). Physical activity can reduce depression levels and can increase the sense of wellbeing among people with mental disorders (Li et al., 2022). Different exercise categories, such as frequency and intensity of exercise, have different degrees of impact on mental health (Duan, 2014). Positive exercise experience helps to enhance the exercise desire, making the students more willing to participate in the exercise actively, whereas negative exercise experience suppresses the exercise intention, weakens the intensity of the exercise motivation, and limits the implementation of the plan (Dong and Zhang, 2016). Both the intensity of physical exercise and the duration of physical exercise can have a significant impact on subjective and psychological wellbeing. The greater the intensity of physical exercise, the stronger the subjective happiness (Li and Liu, 2014).

Most scholars believe that participating in physical activities will have an impact on emotional experiences. Small- and medium-intensity physical activity have a significant impact on individual positive emotional experiences. Appropriate exercise intensity can improve the positive emotional state and reduce the negative emotional state, which has an obvious effect on improving the overall level of positive wellbeing and reducing psychological fatigue (Dong, 2004; Wang and Yan, 2006). Research studies showed that intensive activities can increase happiness in college students, which in turn eliminates psychological disturbance (Lin and Xu, 2020). Leisure sports can help college students to effectively eliminate fatigue, but their effects on improving positive wellbeing and distress are not obvious (Meng, 2015). Currently, there are many studies on physical activity and subjective exercise experience targeting various groups, including children and adolescents, university students, and older adults (Wang and Zhao, 2018; Smith and Hanni, 2019; He et al., 2022). However, the question of whether the effects can be generalized to physically vulnerable college students is still unclear.

The COVID-19 pandemic and associated lockdowns increased the prevalence of anxiety and depressive disorders worldwide, people have less opportunity to participate in exercise (Rahim et al., 2021; Lokman and Bockting, 2022). However, doing some physical activity had lower risks for severe COVID-19 outcomes, including death, than those who were consistently inactive (Sallis et al., 2021). Mandatory physical education (PE) for college students showed tremendous changes among them, especially in Wuhan city. All the PE classes switched to online mode. TikTok, YouTube, and other apps were a few sources of reference for the practice of physical exercise during the COVID-19 outbreak (Vancini et al., 2021). However, the teaching objectives are difficult to achieve due to the limitation of equipment and field. At the same time, the potential burden on psychological health has appeared (Burhamah et al., 2020). In the spring and winter of 2021, although the COVID-19 confirmed cases had significantly declined and it began to resume normal PE, the physical fitness of college students continues to decline compared with aforetime. However, it is unclear how students' subjective exercise experience has changed since the outbreak of the pandemic.

Understanding subjective exercise experience and its predictors carry essential implications for PE in the post-COVID-19 era. Currently, there is scarce evidence that focuses on physically vulnerable college students, which does not mention psychological symptoms (anxiety and depression) that determine their subjective exercise experience and psychological wellbeing. If college students experienced high positive wellbeing after participating in the PE, it will motivate their insistence to exercise, especially for those physically vulnerable college students. The present study used latent profile analysis (LPA) to explore the latent class of subjective exercise experience among physically vulnerable college students who scored 59 points or less on tests under the National Student Physical Health Standard (Zhou et al., 2008).

## Methods

### Participants and procedure

The participants (*N* = 451) were first-year college students from Hubei Province who (i) failed National Student Physical Fitness Test; (ii) were enrolled in the compulsory physical training course; and (iii) were without chronic illness. The study was conducted between March 2020 and September 2021, and three samples were collected in March 2020 (*N* = 127), March 2021 (*N* = 118), and November 2021 (*N* = 206), respectively. Students of compulsory physical training courses voluntarily participated in the study at the beginning of each semester. First, details of the research project were briefed by the instructor of the training course, and the ethical issues (e.g., confidentiality, voluntary participation, and participation that were not related to course grades) were highlighted. Then, online informed consent forms were sent to those who expressed an interest in the research project before accessing the battery of questionnaires through the embedded link to the survey platform (Wenjuanxing). The whole survey took ~15 min to complete. Table 1 summarizes the demographics of the three samples.

**Table 1:** Descriptive statistics of samples.

	**Gender**			**Age**		**BMI** ^**#**^			
**Semester**	***N***	**Men**	**Women**	***M***	***SD***	**Underweight**	**Healthy**	**Overweight**	**Obesity**
2020 Spring	127	72	55	18.76	0.916	20 (15.75%)	86 (67.72%)	16 (12.60%)	2 (1.57%)
2021 Spring	118	26	92	18.54	0.772	22 (18.64%)	70 (59.32%)	18 (15.52%)	6 (5.08%)
2021 Winter	206	77	129	18.15	0.658	38 (18.45%)	122 (59.22%)	34 (16.50%)	9 (4.37%)
Total	451	175	276	18.42	0.81	82 (18.18%)	278 (61.64%)	68 (15.08%)	17 (3.77%)

^♯^According to BMI formula, weight (in kg) is divided by height (in m sq). Underweight: below 18.5 kg. Normal or healthy weight: 18.5-24.9. Overweight: 25-29.9 kg. Obese: 30 kg and above.

### Measures

#### Demographics

The participants reported their age, gender, weight, and height. According to the BMI formula, weight (in kg) is divided by height (in m sq) and categorized into the following four groups: underweight (<18.5), normal or healthy weight (18.5–24.9), overweight (25–29.9), and obese (>30).

#### Subjective exercise experiences

The Subjective Exercise Experiences Scale (SEES) was used to measure the emotional responses after attending compulsory physical training (MeAuley and Courneya, 1994). SEES consists of 12 items in total with three subscales that assessed three dimensions of subjective exercise experience: positive wellbeing, psychological distress, and fatigue. The participants responded on a 6-point Likert scale, ranging from “1 = completely incompatible” to “6 = completely in line”. A higher score of positive wellbeing reflected better exercise experience whereas a higher score of psychological distress and fatigue indicated more negative subjective exercise experience. Cronbach's α value of three subscales were 0.86 (positive wellbeing), 0.90 (psychological distress), and 0.89 (fatigue).

#### Anxiety symptoms

Anxiety symptoms in the past 2 weeks were measured by the Chinese version of the 7-item Generalized Anxiety Disorder (GAD-7) (Spitzer et al., 2006). The participants reported their anxiety symptoms on a 4-point Likert scale ranging from “0 = not at all” to “3 = nearly every day”. Higher scores indicated more severe anxiety symptoms. Probable anxiety was determined by scores at or exceeding 10 (Plummer et al., 2016). Cronbach's α in this study was 0.91.

#### Depressive symptoms

The Chinese 9-item Patient Health Questionnaire (PHQ-9) was adopted to measure depressive symptoms in the past 2 weeks (Yeung et al., 2008). PHQ-9 was a 4-point Likert scale ranging from “0 = not at all” to “3 = nearly every day”, with a higher total score (range = 0–27) indicating more severe depressive symptoms. Probable depression was reflected by the cutoff total score at or exceeding 10 (Yeung et al., 2008). Cronbach's α in this study was 0.83.

### Analytic plan

First, descriptive statistics and zero-order correlations between variables were tested using Statistical Package for Social Sciences (SPSS version 26.0, Chicago, IL, USA). Next, LPA was conducted to explore the latent class of subjective exercise experience using two R-packages: tidyLPA and Mclust (Rosenberg et al., 2019). LPA is a robust and commonly used mixture model for identifying latent profiles. Akaike information criterion (AIC), Bayesian Information Criteria (BIC), Entropy, and Bootstrap Likelihood Ratio Test (BLRT) were used to evaluate 1- to 9-class solutions. A significant increase in the model fit of the k-class model compared with the k-1 class model was indicated by the significant *p*-value of BLRT. We excluded solutions with small classes (<5% of the sample size) because they could be unstable and hard to replicate (Nylund-Gibson and Choi, 2018). Fit statistics, interpretability, and theoretical relevance were all used to make the final decision. Choice of best-fitting solutions considered fit indices, interpretability, explanatory relevance, and theoretical coherence. Lastly, multinomial logistic regression was performed in SPSS to determine potential predictors that were associated with the latent class membership. Odds ratios (ORs) and 95% confidence intervals (Cis) were reported for each predictor entered into the model.

## Results

### Descriptive statistics

Three samples were collected since the COVID-19 pandemic. We defined three stages of COVID-19 based on the report of The National Health Commission of China. The report mentioned that COVID-19 prevention and control in China can be divided into four stages since 2020. The first stage is the emergency containment stage. We called the second phase the exploration phase of regular epidemic prevention and control. The third stage, named the “dynamic zero clearance”, was initiated in August 2021, characterized by whole-chain prevention and control. The three-wave data collection in the current study, namely, Spring 2020, Spring 2021, and Winter 2021, corresponds to the first three stages of epidemic prevention and control in China. The first sample (*N* = 127, 56.69% men) was collected at the emergency containment stage in March 2020. The majority of the participants (67.72%) were healthy, followed by 15.75% of patients who were underweight, 12.6% of those who were overweight, and 1.57% of those who were obese. The second sample (*N* = 118, 22.23% men) was collected 1 year after the outbreak of the pandemic in the exploration phase of regular epidemic prevention and control in March 2021. Participants within the healthy range BMI declined to 59.32%, while the percentage of individuals who were underweight, overweight, and obese all increased. The third sample (*N* = 206, 37.38% men) was collected in the winter of 2022 at the stage of dynamic zero clearance, and the distribution of each BMI category was comparable between the second and third samples. All participants were first-year undergraduate students who failed the National Student Physical Fitness Test, and there was no duplication among the three samples. Anxiety symptoms were positively associated with psychological distress (*r* = 0.37) and psychological fatigue (*r* = 0.32), while depressive symptoms were found to be significantly associated with all three dimensions of subjective exercise experience (*r* = −0.13 to 0.41). Tables 1, 2 summarize descriptive statistics for the participants and the measurements.

**Table 2:** Means, standard deviations, and correlations of variables.

	**SEES: PosiWel**	**SEES: PsyDis**	**SEES: Fatigue**	**GAD-7_total**	**PHQ-9_total**	**Age**	**BMI**
SEES: PosiWel	1						
SEES: PsyDis	−0.249^**^	1					
SEES: Fatigue	0.005	0.565^**^	1				
GAD7: Total	−0.083	0.374^**^	0.322^**^	1			
PHQ9: Total	−0.103^*^	0.410^**^	0.351^**^	0.732^**^	1		
Age	0.017	0.001	−0.014	−0.032	0	1	
BMI	0.034	0.005	−0.011	−0.03	−0.012	0.03	1
M	16.84	9.44	13.50	4.25	4.25	18.42	22.06
SD	3.99	4.23	4.98	3.77	3.74	0.81	4.43

* *p* < 0.05.

** *p* < 0.01 (two-tailed).

### Profiles and prevalence of subjective exercise experience

**Figure 1:**
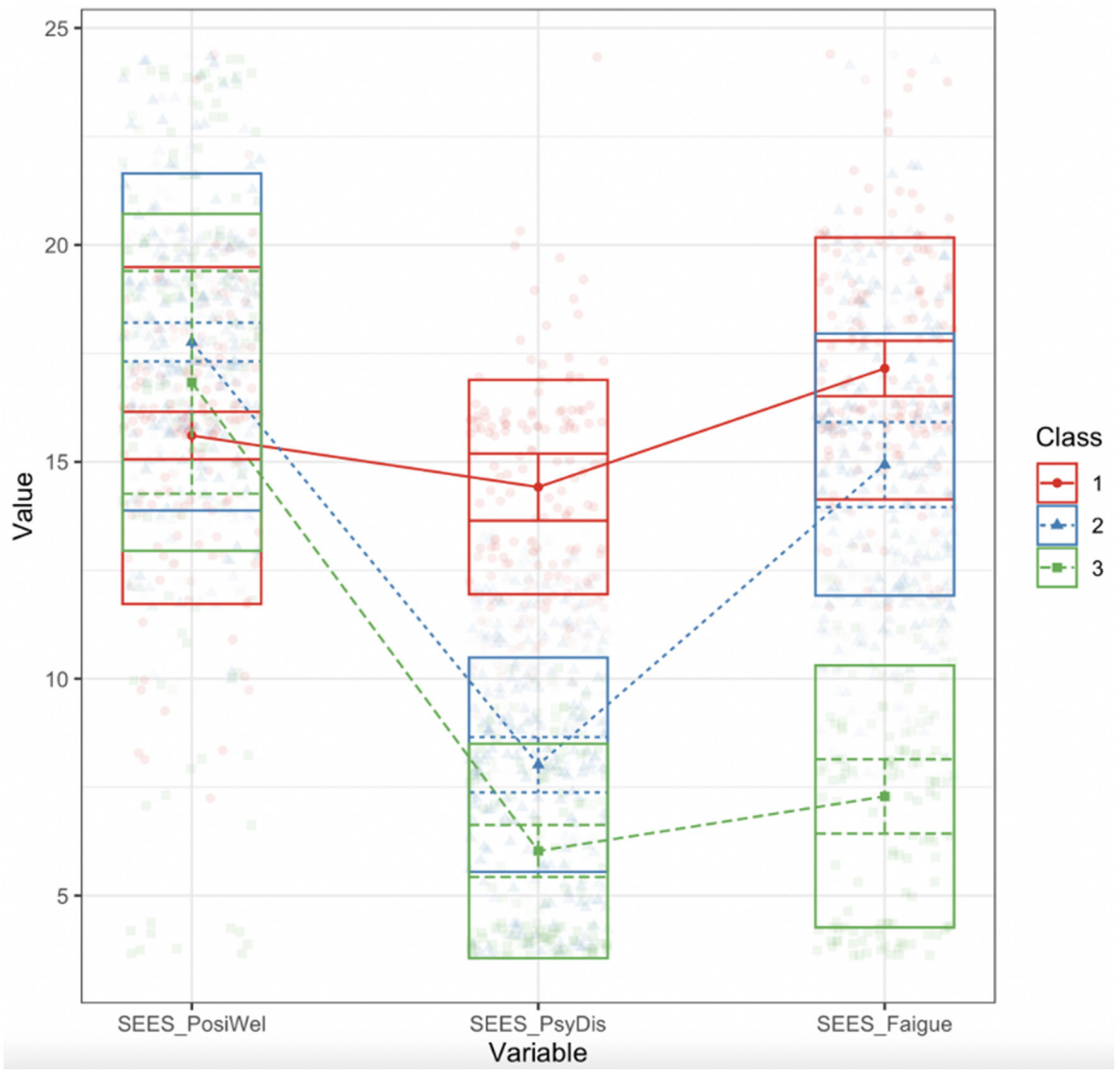
The mean factor score of the subjective exercise experience (SEES) by class. PosiWel: Psychological wellbeing. Fatigue: Psychological fatigue. PsyDis: Psychological distress. Class 1 was the “negative experience group” characterized by low positive wellbeing, high psychological distress, and high psychological fatigue. Class 2 was the “fatigue” group characterized by high positive wellbeing, low psychological distress, and high psychological fatigue. Class 3 was the “positive experience group” characterized by high positive wellbeing, low psychological distress, and low psychological fatigue.

One- to nine-class solutions for subjective exercise experience were examined (as shown in Table 3). Consistently decreased information criteria (AIC and BIC) indicated improved model fit with increased class number. The results of the BLRT were significant in up to eight classes, which compared the n-class solution with the *n* – 1 class solution. However, from the 4-class solution, the solution started to include classes with low prevalence (≤5%), which suggested a marginally stable/unstable class distribution. Therefore, the 3-class solution was selected for the subjective exercise experience of physically unfit students. The mean factor score of the subjective exercise experience (SEES) by class is shown in Figure 1. Three latent-class profiles were defined in terms of performance in the three dimensions of the subjective exercise experience. Class 1 (30.82%) was the “negative experience group” characterized by low positive wellbeing, high psychological distress, and high psychological fatigue. Class 2 (41.91%) was the “fatigue” group, which exhibited high positive wellbeing, low psychological distress, and high psychological fatigue. Class 3 (27.27%) was the “positive experience group” characterized by high positive wellbeing, low psychological distress, and low psychological fatigue. Descriptive statistics of age, gender, BMI, probable anxiety, probable depression, and pandemic stage by class were demonstrated in Table 4.

**Table 3:** Fit indices for different models with a number of clusters ranging from 1 to 9.

**Classes**	**AIC**	**BIC**	**Entropy**	**Percentage small**	**BLRT**
1	7,846.04	7,870.71	1	100%	
2	7,670.88	7,712	0.75	38%	0.01
3	7,590.9	7,648.46	0.71	27%	0.01
4	7,446	7,520	0.8	5%	0.01
5	7,407.92	7,498.37	0.77	4%	0.01
6	7,362.03	7,468.93	0.82	4%	0.01
7	7,312.76	7,436.1	0.87	3%	0.01
8	7,260.87	7,400.65	0.9	2%	0.01
9	7,262.98	7,419.22	0.88	2%	0.28

**Table 4:** Descriptive statistics of age, gender, BMI, probable anxiety, probable depression, and pandemic stage by class.

	**Number (%)**			
**Variables**	**Class 1**	**Class 2**	**Class 3**	**Total**
**Gender**				
Men	45 (32%)	75 (40%)	55 (45%)	175 (39%)
Women	94 (68%)	114 (60%)	68 (55%)	276 (61%)
**BMI**				
Underweight	29 (21%)	28 (15%)	25 (21%)	82 (18%)
Overweight	24 (17%)	30 (16%)	14 (12%)	68 (15%)
Obesity	8 (6%)	6 (3%)	3 (3%)	17 (4%)
Healthy	78 (56%)	122 (66%)	78 (65%)	278 (63%)
**Probable anxiety**				
Yes	15 (11%)	15 (8%)	6 (5%)	36 (8%)
No	124 (89%)	174 (92%)	117 (95%)	415 (92%)
**Probable depression**				
Yes	22 (16%)	18 (10%)	3 (2%)	43 (10%)
No	117 (84%)	171 (90%)	120 (98%)	408 (90%)
**Pandemic stage**				
2020 Spring	31 (22%)	67 (35%)	29 (24%)	127 (28%)
2021 Spring	47 (34%)	44 (23%)	27 (22%)	118 (26%)
2021 Winter	61 (44%)	78 (41%)	67 (55%)	206 (47%)

Class 1 was the “negative experience group” characterized by low positive wellbeing, high psychological distress, and high psychological fatigue. Class 2 was the “fatigue” group, characterized by high positive wellbeing, low psychological distress, and high psychological fatigue. Class 3 was the “positive experience group” characterized by high positive wellbeing, low psychological distress, and low psychological fatigue.

### Multinominal regression

To determine potential predictors associated with the latent class membership, age, gender (men vs. women), BMI (underweight, overweight, obesity, and healthy), probable anxiety (yes vs. no), probable depression (yes vs. no), and pandemic stage (2020 spring, 2021 spring, and 2021 winter) were entered into multinominal regression (as shown in Supplementary material). The results showed that being male (OR: 0.496, 95% CI: 0.279–0.880), those who did not have probable anxiety (OR: 0.120, 95% CI: 0.025–0.576), and those who were associated with an increased likelihood of being in the positive exercise experience group (class 3) rather than the negative experience group (class 1). Whereas, having probable depression and being in the cluster of 2020 spring (OR: 2.143, 95% CI: 1.168–3.931) were associated with an increased risk of being in the fatigue group (class 2) rather than the positive experience group (class 3).

## Discussion

After the outbreak of the pandemic, universities postponed the spring semester, and all classes were converted to online mode, including the PE. The mental health of college students was not optimistic amid the pandemic. For college students, extended holidays, long-term stay at home, and unable to go to school to participate in social activities can increase feelings of anxiety and depression (Chang et al., 2020). Acquisition of positive wellbeing through physical exercise can improve depressive symptoms (Wang, 2021). Even more, moderate-intensity exercise could cut down antidepressant use (Vancampfort et al., 2017).

Research show that even though students understand the importance of PE, they hardly take action because of the limited space at home and the teacher cannot supervise them (Lin and Xu, 2020). This can partially explain our finding that students in the BMI health group gradually decreased since the outbreak of the COVID-19 pandemic, while the other three groups, namely, obese, overweight, and underweight groups increased. Students' physical fitness continues to decline after the end of the pandemic, and this phenomenon reminds us to pay attention to PE.

Although past research has supported the three dimensions of subjective exercise experience (MeAuley and Courneya, 1994), profiles of subjective exercise experience among physically vulnerable students have yet to be established. Men experienced more positive subjective exercise experience compared with women, characterized by high positive wellbeing, low psychological distress, and fatigue. This finding is consistent with the findings of the gender differences in physical activity that men have more healthy physical exercise behavior than women to achieve the same performance (Wang and Yan, 2022). The reason for this result may be that, if female students want to do the same amount of exercise as males students, they have to pay double, or even many times the effort, which may reduce their positive subjective exercise experience. At the same time, the somatic reaction after finishing the exercise also aggravates psychological distress and fatigue (Tao, 2013). Therefore, gender differences should be taken into account in PE, specifically to adjust physical demands and exercise intensity for male and female students. Depressive symptoms are more likely to trigger the psychological fatigue component of the subjective exercise experience. We should not blindly pursue high-intensity and long-time exercise since exercise intensity and exercise time can be maintained at a moderate level.

Several limitations of the present study warn concerns. First, subjective exercise experience was measured by the self-reported instrument. Future studies may consider using both subjective and objective measures to cross-validate physical exercise experience. Second, a convenient sampling method was used, which impeded the generalization of the research findings. Finally, the cross-sectional study design cannot verify the potential reverse causation. Therefore, a longitudinal design is encouraged in future studies.

## Conclusion

Using LPA, we identified three profiles of emotional response toward physical exercise among physically vulnerable students in terms of positive wellbeing, psychological distress, and psychological fatigue. The prevalence of the three profiles had similar distribution among the students. Gender, depression, and anxiety were all found to predict class membership, which was consistent with previous literature highlighting the poor exercise experience of women and individuals with depressive or anxiety symptoms (Ivancic et al., 2016). The hallmark of the present study is that it tested the three salient stages of COVID-19 in China concerning reports of The National Health Commission of China, and supported that students in the emergency containment stage were more likely to experience exercise fatigue compared with students in the dynamic zero clearance stage. The present study has called for attention to these factors when implementing programs for physically vulnerable college students in PE.

## Data availability statement

The raw data supporting the conclusions of this article will be made available by the authors upon request, without undue reservation.

## Ethics statement

The studies involving human participants were reviewed and approved by Ethics Committee of Zhongnan University of Economics and Law. The patients/participants provided their written informed consent to participate in this study.

## Author contributions

Conceptualization and writing—original draft preparation: JL and HL. Methodology, software, formal analysis, investigation, resources, data curation, supervision, project administration, and funding acquisition: JL. Validation and writing—review and editing: MX, HH, and HL. Visualization: MX. All authors have read and agreed to the published version of the manuscript.
