# Supplementary material for: A latent profile analysis of subjective exercise experiences among physically vulnerable college students and psychiatric symptoms correlates during three phases of the COVID-19 pandemic in Wuhan, China
Source: Front Psychol. 2023 Mar 30;14:1118489. doi: 10.3389/fpsyg.2023.1118489 (PMC10102910; doi:10.3389/fpsyg.2023.1118489)
Supplement: Supplementary file 1 [file Table_1.docx]

**Supplementary Material**

Multinomial logistic regression of predictors associated with class membership.

|  | Multinominal Regression  OR (95%CI) | | | |
| --- | --- | --- | --- | --- |
| **Predictors** | Class 1 | *P* value | Class 2 | *P* value |
| **Age** | 0.988(0.702-1.389) | 0.943 | 1.007(0.738-1.375) | 0.965 |
| **Gender** |  |  |  |  |
| Female | 1 [Reference] | - | 1 [Reference] |  |
| Male | 0.496(0.279-0.88) | 0.017 | 0.598(0.355-1.006) | 0.053 |
| **BMI** |  |  |  |  |
| Healthy | 1 [Reference] |  | 1 [Reference] |  |
| Underweight | 1.092(0.572-2.087) | 0.789 | 0.7(0.373-1.311) | 0.265 |
| Overweight | 1.844(0.839-4.051) | 0.128 | 1.581(0.76-3.287) | 0.220 |
| Obesity | 3.406(0.796-14.576) | 0.099 | 1.504(0.332-6.805) | 0.596 |
| **Probable anxiety** |  |  |  |  |
| Yes | 1 [Reference] |  | 1 [Reference] |  |
| No | 0.12(0.025-0.576) | 0.008 | 0.719(0.228-2.268) | 0.574 |
| **Probable depression** |  |  |  |  |
| Yes | 1 [Reference] |  | 1 [Reference] |  |
| No | 0.697(0.211-2.3) | 0.554 | 0.19(0.04-0.907) | 0.037 |
| **Pandemic stage** |  |  |  |  |
| 2021 Winter | 1 [Reference] |  | 1 [Reference] |  |
| 2020 Spring | 1.409(0.711-2.792) | 0.326 | 2.143(1.168-3.931) | 0.014 |
| 2021 Spring | 1.736(0.92-3.275) | 0.089 | 1.277(0.688-2.368) | 0.439 |

Note. Class 3 was the reference group. Class 1 was the “negative experience group”. Class 2 was the “fatigue” group. Class 3 was the “positive experience group”.
